# Effects of equal-volume resistance training with different training frequencies in muscle size and strength in trained men

**DOI:** 10.7717/peerj.5020

**Published:** 2018-06-22

**Authors:** Paulo Gentil, James Fisher, James Steele, Mario H. Campos, Marcelo H. Silva, Antonio Paoli, Jurgen Giessing, Martim Bottaro

**Affiliations:** 1 College of Physical Education and Dance, Universidade Federal de Goiás, Goiania, GO, Brazil; 2 School of Sport, Health, and Social Sciences, University of Southampton, Southampton, United Kingdom; 3 Department of Biomedical Sciences, University of Padova, Padova, Italy; 4 Institute of Sport Science, Universität Koblenz-Landau, Landau, Germany; 5 Faculdade de Educação Física, Universidade de Brasília, Brasilia, DF, Brazil

**Keywords:** Resistance training, Skeletal muscle, Lean muscle mass, Muscle adaptation

## Abstract

**Background:**

The objective of the present study was to compare the effects of equal-volume resistance training (RT) performed with different training frequencies on muscle size and strength in trained young men.

**Methods:**

Sixteen men with at least one year of RT experience were divided into two groups, G1 and G2, that trained each muscle group once and twice a week, respectively, for 10 weeks. Elbow flexor muscle thickness (MT) was measured using a B-Mode ultrasound and concentric peak torque of elbow extensors and flexors were assessed by an isokinetic dynamometer.

**Results:**

ANOVA did not reveal group by time interactions for any variable, indicating no difference between groups for the changes in MT or PT of elbow flexors and extensors. Notwithstanding, MT of elbow flexors increased significantly (3.1%, *P* < 0.05) only in G1. PT of elbow flexors and extensors did not increase significantly for any group.

**Discussion:**

The present study suggest that there were no differences in the results promoted by equal-volume resistance training performed once or twice a week on upper body muscle strength in trained men. Only the group performing one session per week significantly increased the MT of their elbow flexors. However, with either once or twice a week training, adaptations appear largely minimal in previously trained males.

## Introduction

Designing resistance training (RT) programs involves the manipulation of numerous variables that interact with each other (e.g., number of sets, repetitions, rest intervals, etc.), which can have a large influence on the program outcomes (Paoli, 2012; Gentil et al., 2017a). Among them, training frequency has recently received increased attention and some authors consider it one of the most effective strategies to progress a resistance training program (Dankel et al., 2017). Considering that lack of time is a common barrier to exercise adoption (Trost et al., 2002), identifying the minimal frequency of RT to optimize adaptation is of importance. In this regard, condensing exercise sessions into fewer days, while still performing an equal volume, might be a promising strategy, since it would reduce the number of days required and also reduce the total time spent, considering the time necessary for preparation and transport. Though it is argued that it might be beneficial to train with higher frequencies to continue to produce adaptations (Dankel et al., 2017), this might be challenging for most participants. Thus it is important to evaluate the effectiveness of lower-frequency training programs.

Confirming the feasibility of this strategy, previous studies have shown that performing RT only once a week increased muscle size and strength in untrained people (Graves et al., 1990; Carpenter et al., 1991; Gentil et al., 2015). However, little is known about the benefits of performing RT at low training frequencies in the gains of muscle size and strength in resistance-trained individuals. The American College of Sports Medicine recommends that trained participants use a frequency of four to five days per week for the purpose of increasing muscle size and strength (ACSM, 2009), although it is not clear if this frequency is referring to the number of sessions performed or the number of times a given muscle is trained per week. The recommendation of higher training frequencies for trained people is supported by the results of meta-analyses which suggest that experienced individuals might benefit from training a muscle group multiple times per week (Rhea et al., 2003; Peterson, Rhea & Alvar, 2004; Schoenfeld, Ogborn & Krieger, 2016; Grgic et al., 2018). However, previous studies in bodybuilders reported that they usually perform split routines training each muscle group only once per week (Hackett, Johnson & Chow, 2013; Gentil et al., 2017b).

Increases in muscle size may in fact plateau relatively early after initiation of a RT intervention suggesting trained persons have limited capacity to further increase muscular size (Counts et al., 2017). However, this may be due to the attenuated anabolic response to RT in trained individuals, and Dankel et al. (2017) have recently argued that there may be a benefit for trained persons to perform greater frequencies of training. Given that there is an increased difficulty in achieving significant results in trained individuals in response to a RT stimulus, as evidenced through attenuated muscle protein synthesis (MPS), higher training frequencies might allow for more frequent MPS rises and thus a greater MPS area under the curve (Dankel et al., 2017). However, there is presently a lack of studies testing this idea. We are aware of only two studies comparing the gains in muscle size and strength between different training frequencies in trained participants (McLester, Bishop & Guilliams, 2000; Schoenfeld et al., 2015) and the results are conflicting.

When analyzing experienced weightlifters, McLester, Bishop & Guilliams (2000), concluded that three days per week of equal-volume resistance training was superior to one day per week for bringing about strength gains. However, it is important to note that the group that trained once per week were apparently stronger at baseline, which may have influenced the comparisons. Moreover, the use of indirect measures to evaluate changes in body composition (skinfolds and circumferences) may have limited the capacity for evaluating muscle hypertrophy. Later, Schoenfeld et al. (2015) compared the effects of performing one or three sessions per muscle group per week in resistance trained men. According to the results, higher frequency resulted in greater increases in the MT of elbow flexors; however, no differences were observed for elbow extensor or vastus lateralis muscle thickness. Similarly, statistically significant differences between groups were not noted for 1RM bench press and back squat.

If trained individuals can obtain similar results with lower training frequencies, this could be a valuable strategy for prescribing RT programs for people with time constraints. Therefore, the information provided by the present study would be valuable for conserving training time and encourage participation whilst optimizing adaptation, as well as adding to the existing body of knowledge on training variables. With this in mind, the purpose of this study was to compare the effects of training one or two times per week on strength and muscle size in trained college-aged men, while holding the total number of sets per week constant. The hypothesis of the study is that training one or two days per week would result in similar gains in muscle size and strength.

## Methods

### Experimental approach to the problem

The participants were pair matched by baseline elbow flexor peak torque (PT) and then randomly assigned into one of two groups: Group 1 (G1, *n* = 8) trained upper body once a week and Group 2 (G2, *n* = 8) trained twice a week. G1 and G2 performed the same exercises, with the same number of sets per week. All exercises were performed with three sets of eight to 12 repetitions performed to momentary concentric failure as previously defined (Steele et al., 2017). Before and after the 10 week training period, participants were evaluated for elbow flexor MT and elbow extensor and flexor PT.

### Subjects

Twenty male college students volunteered to participate in the study. Volunteers were invited among those engaged in resistance training classes at the University. This sample size was justified by a priori power analysis based on previous work by Schoenfeld et al. (2015) with a target effect size difference of 0.6, alpha of 0.05 and power of 0.80. The criteria for entering the study included being at least 18 years old, having at least 12 months of previous RT experience and having been practicing RT with direct supervision uninterruptedly for the previous six months, and being free of health problems that could prevent the participation in the study. To be included in the analyses, subjects had to attend at least 80% of the training sessions (Gentil & Bottaro, 2013). The volunteers were instructed to not change their nutritional habits during the study period, all of them verbally confirmed that they maintained their diet throughout the trial period and no relevant change was reported (i.e., becoming a vegetarian, restricting calories, taking nutritional supplements or ergogenic aids, etc.). At the end of the study, 16 subjects met the criteria for entering the analysis (22.3 ± 2.0 years; 177.5 ± 5.1 cm; 80.0 ± 12.4 kg). The exclusions (two in each group) were due to engagement in RT sessions other than the study protocol, changes in nutritional habits (one participant became vegetarian) and/or low training attendance (three participants). All participants had a history of training each muscle group two to three times per week, and all of them have been training each muscle group two times a week in the previous four months.

The volunteers were notified of the research procedures, requirements, benefits and risks before providing written informed consent. The Institutional Research Ethics Committee granted approval for the study (56907716.5.0000.5083) and the study was performed according to the Declaration of Helsinki.

### Muscle thickness

Muscle thickness (MT) of the elbow flexors was measured before and after the 10-week training period using B-Mode ultrasound (Philips-VMI, Ultra Vision Flip, model BF, Amsterdam, Netherlands). The tests were conducted three to five days after the last training session to prevent any swelling from influence measurement. During this time, participants were oriented not to participate in any other exercise sessions or intense activity. All tests were conducted at the right arm, at the same time of the day and the participants were oriented to hydrate normally 24 h before the tests. MT of the elbow flexors was measured according to Bemben’s procedures (Bemben, 2002) and was taken as the distance from the subcutaneous adipose tissue-muscle interface to muscle-bone interface (Abe et al., 2000). A trained technician performed all analyses. Baseline test and retest intraclass correlation coefficient (ICC) for MT of elbow flexors was 0.95.

### Isokinetic peak torque

Unilateral elbow flexion and extension isokinetic peak torque (PT) were tested on a Biodex System 3 isokinetic dynamometer (Biodex Medical, Inc., Shirley, NY, USA). Tests were performed at the dominant side with two sets of four concentric contractions at 60° s^−1^ and 60 s rest between sets. The dynamometer was calibrated prior to each testing session according to manufacturer specifications. Participants were seated on a Scott Bench and the lateral epicondyle of the humerus was used to align elbow rotation to the dynamometer’s lever arm. Volunteers were instructed to perform maximal efforts in all tests, and verbal encouragement was constantly provided by the researchers. During elbow flexion, the forearm remained in a supinated position throughout the test. For elbow extensions, the forearm remained in a neutral position. Baseline test and retest ICC for peak torque were 0.96 for both elbow flexion and extension.

### Resistance training intervention

Participants were divided into two groups. G1 (*n* = 8) trained once a week (Mondays) and G2 (*n* = 8) trained twice a week (Mondays and Thursdays), and their characteristics are presented in Table 1. The participants were allocated into groups in a counterbalanced manner according to their values of elbow flexor PT. Both groups performed the same exercises, with equal number of sets and repetition ranges; therefore, the only difference was that G1 trained each muscle group once per week and G2 trained twice. The following exercises were performed: lat pull down, seated row, barbell bench press, seated chest press, standing barbell biceps curl, Scott bench biceps curl, lying barbell triceps extension and high pulley triceps extension. All exercises were performed with three sets of eight to 12 repetitions to momentary concentric failure (Steele et al., 2017), and were provided with verbal encouragement to maximize intensity of effort. If necessary, loads were adjusted from set to set and between sessions to maintain performance of the desired number of repetitions. All training sessions were closely monitored to ensure effort, repetitions and intensity established by experienced strength and conditioning coaches, since previous research has demonstrated greater gains in supervised vs. unsupervised training sessions (Gentil & Bottaro, 2010). Rest interval between sets and exercises was maintained at 2 minutes.

**Table 1:** Training sessions for Groups 1 and 2.

	Monday	Thursday
Group 1	Lat pull down	xxxx
	Seated row	
	Standing barbell biceps curl	
	Scott bench biceps curls	
	Barbell bench press	
	Seated chest press	
	Lying barbell triceps extensions	
	High pulley triceps extension	
Group 2	Lat pull down	Seated row
	Standing barbell biceps curl	Scott bench biceps curls
	Barbell bench press	Seated chest press
	Lying barbell triceps extensions	High pulley triceps extension

### Statistical analysis

Normality of the data was confirmed using the Kolmogorov–Smirnov test. Data are presented as mean ± standard deviation. Groups were compared using factorial mixed model ANOVA 2 × 2 (Group × Time). When necessary, multiple comparisons with confidence adjustment by the Bonferroni procedure were used for post hoc analysis. Data were considered significant at *P* < 0.05. Within groups, effect size (ES) was calculated using Cohen’s *d* (threshold values were 0.2 for small, 0.5 for moderate and 0.8 for large). Statistical analyses were performed using the Statistical Package for the Social Sciences 17.0 software (SPSS, Chicago, IL, USA).

## Results

**Figure 1:**
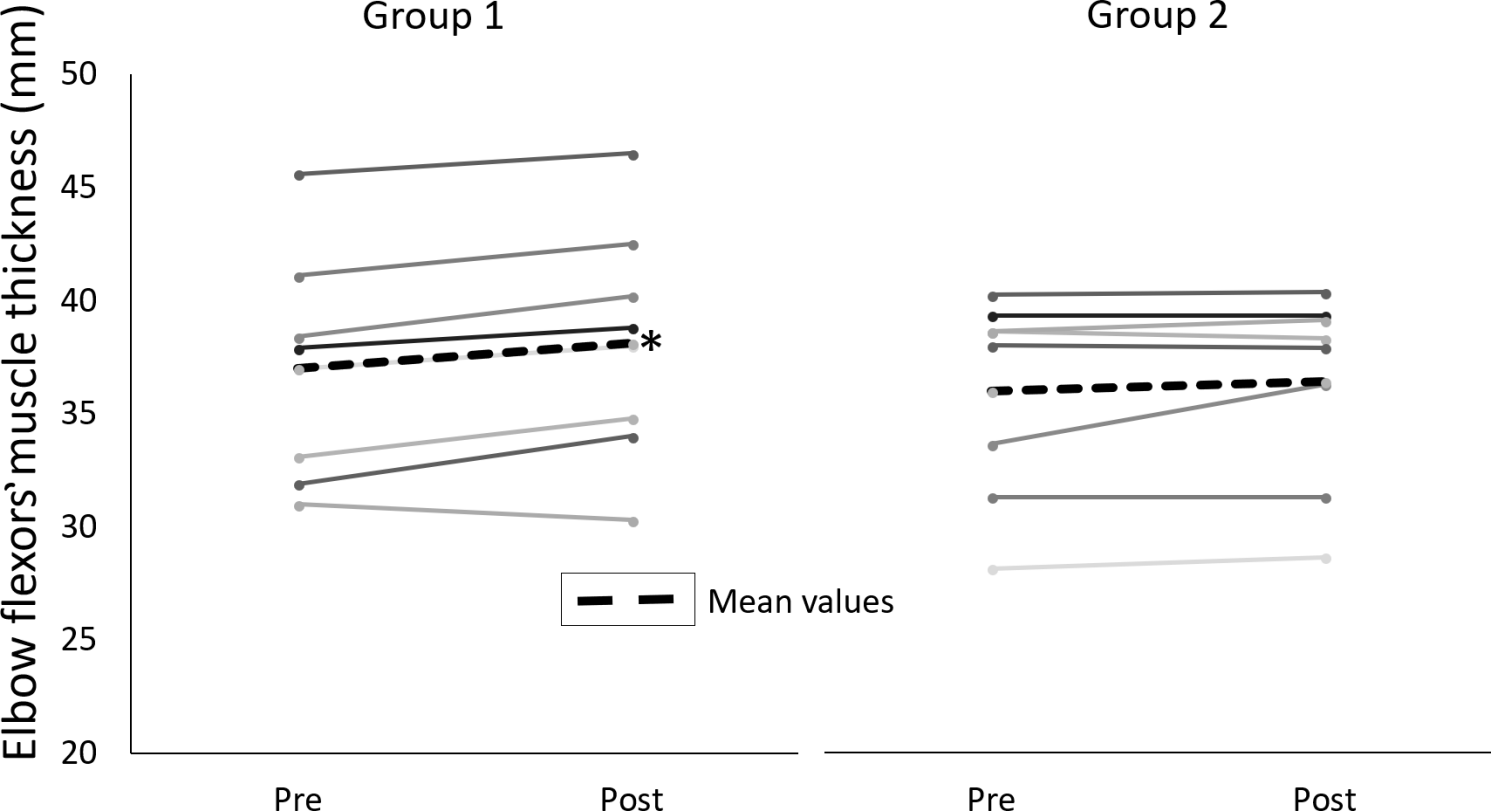
Changes in muscle thickness of elbow flexors. Individual values for pre- and post-training muscle thickness of elbow flexors (mm). Group 1 trained each muscle group once a week and Group 2 trained each muscle group twice per week. * significant difference between pre- and post-values (*p* < 0.05).

Table 2 presents the characteristics of the participants and Table 3 presents the peak torque values. The results for elbow flexor muscle thickness are presented in Fig. 1. There was no significant difference in baseline values for age, height and body mass between G1 and G2. The results of ANOVA did not reveal group by time interactions for any variable, indicating no difference between groups for the changes in any of the muscle size and strength variables. Elbow flexor and elbow extensor PT did not increase significantly for any group. Elbow flexor MT increased significantly only for G1 (3.1% *P* < 0.05).

**Table 2:** Characteristics of the participants in each group (mean ± standard deviation).

	Group 1	Group 2
Age (years)	21.7 ± 2.1	22.8 ± 2
Weight (kg)	78.3 ± 14	81.7 ± 11.3
Height (cm)	176 ± 4.9	178.9 ± 5.2
Resistance training experience (months)	14.4 ± 4.4	16.8 ± 8.8

**Table 3:** Pre and post values for peak torque of elbow flexors and extensors, expressed as mean ±  standard deviation.

	Group 1				Group 2			
	Pre	Post	Delta	ES	Pre	Post	Delta	ES
Elbow flexors’ peak torque (N.m)	66.6 ± 12.1	66 ± 11.6	−0.9%	−0.03	66.7 ± 13.9	67.1 ± 12.7	0.6%	0.02
Elbow extensors’ peak torque (N.m)	57.7 ± 10.2	57.6 ± 5.7	−0.1%	−0.01	55.4 ± 13.1	55.8 ± 11.7	0.9%	0.02

## Discussion

The results of our study showed that there were no differences in performing RT one or two times a week with equal number of sets in trained men. Nonetheless, only the group that trained once per week significantly increased muscle thickness. This is similar to a previous study from our group using a similar training program in untrained participants where there were no differences between groups for increases in muscle strength and size; however, it is important to note that the effect sizes of muscle strength were higher in the group that performed two sessions per week (Gentil et al., 2015).

The results of the present study seem to contradict the results of previous studies in trained men. When analyzing experienced weightlifters, McLester, Bishop & Guilliams (2000) concluded that three days per week of equal-volume resistance training was superior to one day per week for strength gains, suggesting that higher frequency of training may be superior for trained individuals. However, as previously noted, the group that trained at higher frequencies had lower strength levels at baseline. Considering that higher initial values may be related to limited strength increases, this may have influenced the results. More recently, Schoenfeld et al. (2015) compared the effects of exercising each muscle one or three days per week in trained young men and suggested that increased frequency would be beneficial for muscle strength and size. However, the authors highlighted that 16 of the 19 subjects reported training with a split routine on a regular basis, therefore, the novelty factor of changing programs might have influenced results.

Interestingly, all participants of the present study have been training at higher frequencies (exactly the same protocol performed by G1) for at least four months before the study and only those that decreased frequency showed increases in muscle size. For this reason, we cannot rule out that any positive adaptations were due to a variation in training stimuli and not a benefit of reduced frequency per se. This might suggest that changing the usual stimuli may be necessary to bring continued adaptations.

The lack of results in most variables seen in the present study is not surprising. Once an individual is used to a given stimulus, there is a decline in training response, and a plateau occurs far earlier than generally expected (Counts et al., 2017). Previous studies demonstrated that muscle hypertrophy and strength gains in response to resistance training seem to progressively diminish after a few weeks of training (Correa et al., 2013; Nader et al., 2014) and comparisons of muscle size and strength gains between untrained and trained participants showed a reduced response in the latter (Ahtiainen et al., 2003). In order to overcome this plateau, it seems necessary to provide novel stimuli which is usually done by changing load, repetition range and the exercises performed; the results of our study might indicate that changing training frequency may be an effective strategy as well.

Considering that protein synthesis may return to basal levels in a few days after the training session (Chesley et al., 1992; MacDougall et al., 1995; Burd et al., 2012), it has been suggested that higher training frequency may promote a more favorable anabolic balance, increasing long-term results (Dankel et al., 2017) Nevertheless, this was not observed in the present study. In fact, recent studies showed that trained men did not recover neuromuscular capacity four days after a high volume resistance training session (Ferreira et al., 2017b; Ferreira et al., 2017a), suggesting that longer intervals between sessions might be necessary when training with higher numbers of sets (>eight sets per muscle group). Although it is commonly suggested that training must be repeated after two to three days (ACSM, 2009), there are studies reporting positive results in trained men training once a week (Ostrowski et al., 1997) and this approach has been widely used by bodybuilders using split programs (Hackett, Johnson & Chow, 2013).

As lack of time is the most frequently cited barrier to exercise adoption (Trost et al., 2002) using an exercise program that can be performed only once a week may improve adherence in periods where time constraint might be an obstacle to continue training. Moreover, coaches and athletes might consider including variations in training frequency in their training programs in order to overcome plateaus in muscle hypertrophy. In order to gain further insight into time efficiency it would be of interest to test the effectiveness of low training frequency with a low number of sets, which could be compensated by increased intensity of effort. This was successfully employed in older people (Fisher et al., 2014; Barbalho et al., 2017), but we are not aware of similar studies in young trained participants.

This study is not without limitations. First, although the duration of the study was similar to previous studies, 10 weeks may have not been long enough to allow us to find statistical differences both within and between groups. In addition, the small sample size might have affected statistical power. Despite this limitation, the inter-individual variability was not high, except for elbow extensor PT, and analysis of effect sizes provides a good basis for inferring that the results would not be clinically meaningful. Lastly, as Dankel et al. (2017) note, there is a lack of studies examining frequencies higher than three times a week. It remains a possibility that the lack of change in many outcomes for the present study was due to both groups using relatively low frequencies (≤twice a week). Therefore, future research should look to compare both lower (≤twice a week) to higher frequencies (>three days a week).

The study did not have a non-training control group because our purpose was to compare different training frequencies. However, since the study involved trained people that are used to both the training and testing procedures, learning is not expected to influence performance of the tests. It is also important to note that, whilst training frequency changed for each situation, the total training volume was kept constant for both groups; therefore, the duration of the session was longer for the group that trained only one time per week. This should be taken in account, since the increased time demand at a specific session might be a barrier for some people.

## Conclusion

Trained men that are used to training at higher frequencies could benefit from decreasing training frequency when pursuing muscle hypertrophy. Considering that the results seem to be related to an unaccustomed stimulus, coaches and athletes might consider including variations in training frequency, while keeping the number of weekly sets constant, in their training programs in order to overcome plateaus in muscle hypertrophy. Moreover, reducing training frequency may also be an efficient strategy to reduce time commitment without interfering with the results.

##  Supplemental Information

10.7717/peerj.5020/supp-1 Supplemental Information 1 Data of the participants of the study

## Additional Information and Declarations

### Data Availability

The following information was supplied regarding data availability: The raw data are provided in Supplemental Information 1.
